# Chronic disseminated candidiasis manifesting as hepatosplenic abscesses among patients with hematological malignancies

**DOI:** 10.1186/s12879-019-4260-4

**Published:** 2019-07-17

**Authors:** Chien-Yuan Chen, Aristine Cheng, Feng-Ming Tien, Po-Chu Lee, Hwei-Fang Tien, Wang-Huei Sheng, Yee-Chun Chen

**Affiliations:** 1 Division of Hematology, Department of Internal Medicine, National Taiwan University Hospital, No. 7 Chung-Shan South Road, Taipei, 10002 Taiwan; 2 Division of Infectious Disease, Department of Internal Medicine, National Taiwan University Hospital, Taipei, Taiwan; 3 Tai-Cheng Stem Cell Center, National Taiwan University Hospital, Taipei, Taiwan; 4 Graduate Institute of Clinical Medicine, National Taiwan University, Taipei, Taiwan; 5 Department of Surgery, National Taiwan University Hospital, Taipei, Taiwan; 6 Department of Trauma, National Taiwan University Hospital, Taipei, Taiwan; 7 Department of Medicine, National Taiwan University, College of Medicine, No. 7 Chung-Shan South Road, Taipei, 10002 Taiwan

**Keywords:** Chronic disseminated candidiasis (CDC), Hepatosplenic candidiasis, Hematological malignancy, Resolution of image, Survival

## Abstract

**Background:**

The outcomes of deep-seated abscesses attributed to chronic disseminated candidiasis (CDC) in patients with hematological malignancies have rarely been reported in recent years.

**Methods:**

We retrospectively reviewed and analyzed the data of patients with hematological malignancies who received a diagnosis of CDC at a medical center in Taiwan between 2008 and 2013.

**Results:**

Sixty-one patients (32 men and 29 women) were diagnosed with CDC. The median age was 51 years (range: 18–83). The overall incidence of CDC was 1.53 per 100 patient-years in patients with hematological malignancies between 2008 and 2013. The highest incidence of CDC was 4.3 per 100 patient-years for acute lymphoblastic leukemia, followed by 3.6 for acute myeloid leukemia. We detected 3 (4.9%) proven, 13 (21.3%) probable, and 45 (73.8%) possible cases of CDC. A total of 13 patients had positive blood cultures for *Candida* species: *C. tropicalis* (8), *C. albicans* (2), *C. glabrata* (2), and *C. famata* (1).

The median duration of antifungal treatment was 96 days (range: 7–796 days). Serial imaging studies revealed that the resolution rate of CDC was 30.0% at 3 months and 54.3% at 6 months. Five patients (8.2%) had residual lesions that persisted beyond one year. A multivariate analysis of the 90-day outcome revealed that shock was the only independent prognostic factor of 90-day survival in patients with CDC.

**Conclusion:**

The incidence of CDC did not decrease between 2008 and 2013. Patients with acute leukemia had a higher risk of CDC than those with other hematological malignancies. Imaging studies conducted at 6 months after diagnosis revealed that only half of the patients showed complete resolution. CDC requires prolonged treatment, and serial imaging at 6 months interval is suggested. Shock is the only independent prognostic factor of 90-day survival in patients with CDC.

## Background

*Candida* is a common pathogen worldwide and in patients with cancer, high morbidity and mortality are attributed to invasive candidiasis [1, 2]. Chronic disseminated candidiasis (CDC) is a unique clinical manifestation of invasive candidiasis; it usually develops during recovery from neutropenia after chemotherapy and affects organs such as the liver and spleen in patients with acute leukemia [3–6]. The exact pathogenesis of CDC is unknown. Studies have proposed that *Candida* species colonizing the bowel, invade and seed to hepatosplenic sinusoids from the portal splenic bloodstream following chemotherapy-induced mucosal damage [7, 8]. CDC is defined as small, peripheral, target-like abscesses (bull’s-eye lesions) in the liver or spleen demonstrated on computed tomography (CT), magnetic resonance, or ultrasound imaging; it is accompanied by an elevated level of serum alkaline phosphatase. Supporting microbiological characteristics are not required to fulfill the European Organization for Research and Treatment of Cancer (EORTC) criteria for CDC [9, 10].

The reported incidence of CDC has ranged from 2.0 to 7.4% in patients with acute leukemia [5, 6, 11–15]. Rammaert et al. proposed that the incidence has reduced in the last decade, which was possibly due to the prophylactic and preemptive use of antifungal agents [4]. However, in recent years, few studies have analyzed the epidemiology of CDC in patients with hematological malignancies. Although patients with hematological malignancies other than acute leukemia who developed CDC have been observed in our clinical practice and in a previous study [6], the epidemiology of CDC in these patients has rarely been described.

The treatment duration of CDC depends on clinical symptoms and signs, and the resolution of abscesses from imaging studies. The Infectious Diseases Society of America (IDSA) recommends that therapy should be continued until lesions have resolved on repeat imaging, which is usually after several months [16]. The premature discontinuation of antifungal therapy may lead to a relapse of CDC [16]. However, studies conducting serial imaging follow-up of CDC are limited. Thus, in this study, we retrospectively reviewed and analyzed the clinical manifestation, treatment, serial imaging follow-up, and prognostic factors of CDC in 2083 patients with hematological malignancies between 2008 and 2013.

## Methods

The National Taiwan University Hospital (NTUH) is a 2800-bed teaching hospital in the metropolitan area of Taipei that provides both primary and tertiary care. The medical records of patients admitted to the hematological wards of the NTUH from January 1, 2008, to December 31, 2013, were retrospectively reviewed. The inclusion criteria were patients with acute myeloid leukemia (AML), acute lymphoblastic leukemia (ALL), lymphoma, multiple myeloma (MM), chronic myeloid leukemia (CML), myeloproliferative neoplasm (MPN), chronic lymphocytic leukemia (CLL), myelodysplastic syndrome (MDS), or severe aplastic anemia (SAA). Patients with other hematological diseases such as hemolytic anemia, idiopathic thrombocytopenia, hemophilia, or coagulation disorders were excluded. The methods have already been described in our previous studies [17].

Imaging studies using CT and ultrasonography and other examinations were performed if clinically indicated. This retrospective study was approved by the Research Ethics Committee of NTUH (Institutional Review Board No: 20160613132RIND), and informed consent was waived for this analysis based on approval by the Research Ethic Committee, as the data was analyzed anonymously.

### Diagnosis of chronic disseminated candidiasis

CDC was diagnosed according to the EORTC and the Mycosis Study Group consensus criteria to define invasive fungal infection in this study, and CDC cases were categorized as proven, probable, or possible [9, 10].

### Antifungal prophylaxis and antifungal treatment

At NTUH, no antifungal prophylaxis agent was administered to patients with hematological malignancies, except for a nystatin oral suspension, between 2008 and 2013. Patients awaiting allogeneic stem cell transplantation received micafungin for prophylaxis before neutrophil recovery on the transplantation ward. Antifungal treatments were provided according to the local guidelines of the Infectious Diseases Society of Taiwan [18, 19].

### Statistical analysis

We used the Chi-square test for categorical comparisons of the data. Significant predictors in the univariate analysis were included in a forward, stepwise multiple logistic-regression model to identify the most crucial risk factors for 90-day mortality. A Cox proportional hazard analysis was used to determine the relative contribution of various factors to the risk of 90-day mortality. In the multivariate analysis, factors with *P* values < 0.2 in the univariate analysis were selected for the Cox proportional hazard regression analysis. A survival analysis was performed using the Kaplan–Meier estimator and a log-rank test. A *P* value of < 0.05 indicated statistical significance. All statistical analyses were performed using SPSS for Windows (Version 18.0, SPSS Inc., Chicago, Il, USA).

## Results

### Clinical characteristics of 2083 hematological malignancy patients with and without CDC (hepatosplenic microabscesses)

A total of 2083 patients with hematological malignancies between 2008 and 2013 were included in this study, and 61 of these patients were diagnosed with CDC based on clinical and imaging criteria. In total, 968 patients were women, and the median age was 56 years (range: 15–97). A significant proportion of patients (*n* = 607) were aged 65 years-old or more. These elderly patients (≥65 years) had a lower risk of CDC than younger patients (1.5% vs 3.5%, *P* = 0.010). In this cohort, 397 patients underwent allogeneic stem cell transplantation, among whom 26 patients developed CDC (4 before transplantation and 22 after transplantation). Overall, patients who underwent stem cell transplantation had a higher risk of CDC than those who did not undergo transplantation (6.5% vs 2.1%, *P* < 0.001). The other clinical characteristics of the patients are listed in Table 1. The frequency of underlying diabetes mellitus, hepatitis B, and liver cirrhosis was not significantly different among patients with and without CDC.

**Table 1:** Clinical characteristics of 2083 patients with and without chronic disseminated candidiasis (CDC)

	Patients with CDC (*n* = 61, %)	Patients without CDC (*n* = 2022, %)	p
Age			0.010
Age > =65	9 (1.5)	598 (98.5)	
Age < 65	52 (3.5)	1424 (96.5)	
Gender			0.897
Men	32 (2.9)	1083 (97.1)	
Women	29 (3.0)	939 (97.0)	
Allogeneic transplantation			< 0.001
Yes	26 (6.5)	371 (93.5)	
No	35 (2.1)	1651 (97.9)	
Diabetes Mellitus			0.849
Yes	7 (2.5)	276 (97.5)	
No	54 (3.0)	1746 (97.0)	
Hepatitis B carrier			1.000
Yes	9 (3.0)	296 (97.0)	
No	52 (2.9)	1726 (97.0)	
Liver Cirrhosis			1.000
Yes	0 (0.0)	8 (100.0)	
No	61 (2.9)	2014 (97.1)	

**Fig. 1:**
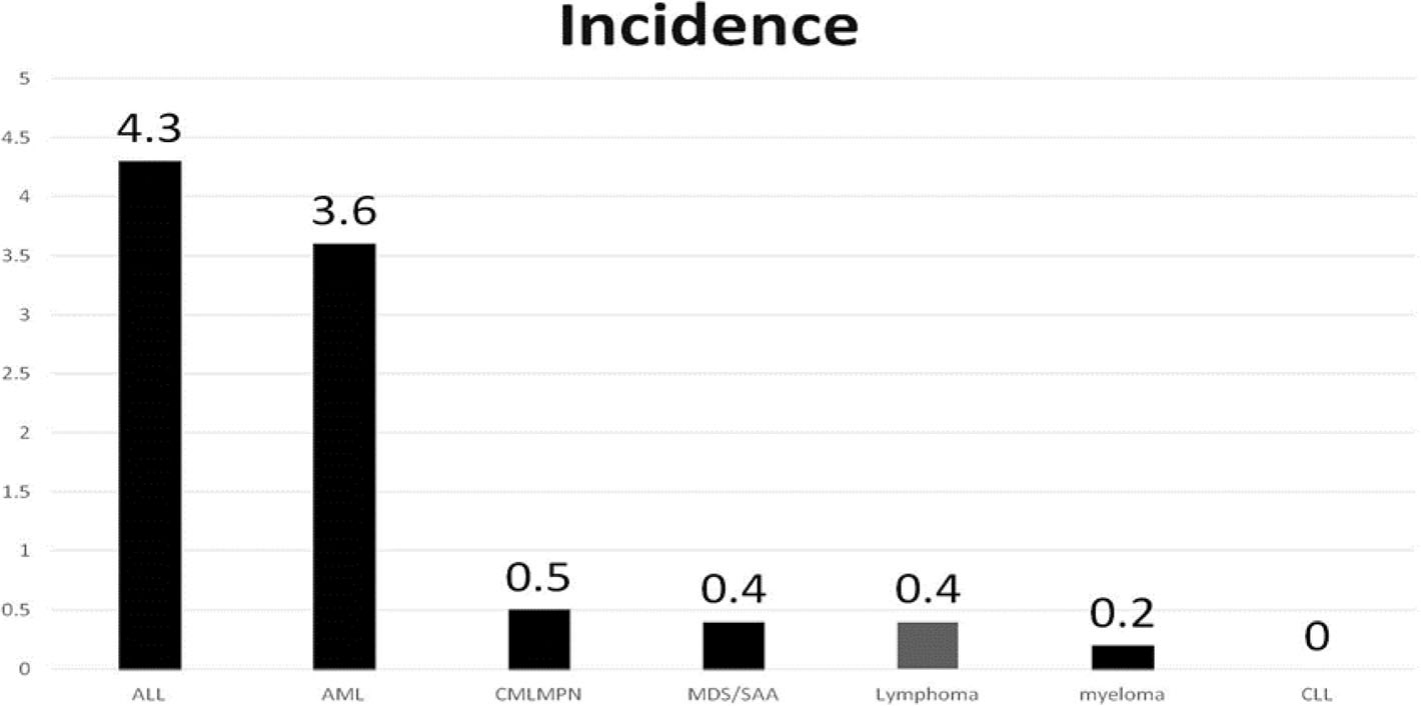
Incidence of hematological malignancies in patients with chronic disseminated candidiasis

The incidence of CDC in patients with hematological malignancies was 1.53 per 100 patient-years between 2008 and 2013 (Fig. 1). The incidence of CDC was highest at 4.3 per 100 patient-years (PYs) in patients with ALL, followed by 3.6 per 100 PYs in patients with AML, 0.5 per 100 PYs in patients with CML or MPN, 0.4 per 100 PYs in patients with lymphoma, 0.4 per 100 PYs in patients with MDS or SAA, 0.2 per 100 PYs in patients with myeloma. No cases of CDC were detected among patients with CLL.

### Clinical manifestation in 61 patients with chronic disseminated candidiasis

In total, 61 patients (32 men and 29 women) were diagnosed with CDC. The median age was 51 years (range: 18–83). The underlying hematological malignancies were AML (*n* = 38), ALL (*n* = 13), lymphoma (*n* = 7), CML/MPN (n = 1), MDS/SAA (n = 1), and myeloma (n = 1). Four patients underwent allogeneic stem cell transplantation before the diagnosis of CDC, and 22 patients underwent allogeneic stem cell transplantation after the diagnosis of the CDC. CDC was detected in all 61 patients either by computed tomography or by abdominal ultrasonography. By imaging studies, CDC was detected in 56 (94.9%) of 59 patients through computed tomography and in 44 (75.9%) of 58 patients through abdominal ultrasonography. Thirty-nine patients had positive findings on both CT and abdominal ultrasound imaging. The most common organs involved were the liver (58/61, 95.1%), spleen (34/61, 55.7%), and incidentally, the kidney (14/61, 23%). Twenty-seven patients underwent liver biopsy, and only 3 patients yielded a confirmatory result (11.1%); candidal hyphae were detected in the pathological specimens of 2 patients and *C. albicans* was recovered by tissue culture in 1 patient. A total of 13 patients had concomitant positive blood cultures yielding *C. tropicalis* (8 patients), *C. albicans* (2 patients), *C. glabrata* (2 patients), and *C. famata* (1 patient), respectively. In this study, we detected 3 (4.9%) proven, 13 (21.3%) probable, and 45 (73.8%) possible cases of CDC.

### Antifungal treatment and resolution of chronic disseminated (hepatosplenic) candidiasis by imaging

A variety of antifungal agents were administered to patients with CDC (Table 2). Thirty-five (57.3%) patients received fluconazole and 9 (14.8%) received amphotericin B as their initial antifungal agent. The median duration of antifungal treatment was 96 days (range: 7–796 days). The median interval between serial imaging studies was 187 days (range: 1–1274). Each patient received a median number of 3.5 CT follow-up studies (range: 0–12) and a median number of 3.3 ultrasound follow-up studies (range: 0–8). All patients underwent at least 3 imaging studies, except for 2 patients due to premature deaths: 1 patient died 11 days after the diagnosis of CDC, and the other patient had persistent lesions at the second CT scan and died 40 days after the diagnosis of CDC. At 3-months following the diagnosis of CDC, 12 of 40 patients (30.0%) had resolution of their hepatosplenic or renal abscesses on imaging, and 28 of 40 patients (70.0%) had residual abscesses. At 6-months, 19 of 35 patients (54.3%) showed complete resolution of abscesses on imaging, and 16 of 35 patients (45.7%) had residual abscesses. Overall, 35 of 61 patients (57.3%) had documented resolution of CDC in this study. The median time to resolution of microabscesses was 161 days (range: 16–1274 days). Five of 61 patients (8.2%) had persistent residual CDC on imaging studies beyond one year. Time to documented resolution of CDC by imaging was analyzed using the Kaplan–Meier method (Table 2). Neutropenia at the diagnosis of CDC (*P* = 0.005) was associated with a more rapid resolution of CDC in imaging studies. Sixteen (84.2%) of 19 patients recovered from neutropenia, but 3 (15.8%) patients died within 90 days.

**Table 2:** Univariate analysis of factors of the resolution of CDC in imaging studies at 6 months

Factor	Residual lesions (*n* = 26)	Resolution (*n* = 35)	*p* value
Gender			0.807
Man	14	18	
Woman	12	17	
Age			0.525
< 65 years	24	28	
≧65 years	2	7	
Acute leukemia			0.807
Yes	21	30	
No	5	5	
Hypertension			0.550
Yes	2	8	
No	24	27	
Diabetes mellitus			0.725
Yes	4	5	
No	22	30	
Hepatitis B carrier			0.808
Yes	1	6	
No	25	29	
Hepatitis C			0.990
Yes	0	2	
No	26	33	
Proven CDC			0.080
Yes	11	5	
No	15	30	
Involved more than 2 organs			0.069
Yes	14	19	
No	12	16	
High dose chemotherapy			0.459
Yes	12	15	
No	14	20	
Preceding HSCT			0.336
Yes	3	2	
No	23	33	
Remission status			
Yes	7	15	
No	19	20	
Neutropenia at diagnosis of CDC			0.005
Yes	5	14	
No	21	21	
Thrombocytopenia at diagnosis of CDC			0.750
Yes	3	6	
No	23	29	
Adequate initial antifungal			0.115
Yes	25	34	
No	1	1	

### Mortality at 90 days in 61 patients with chronic disseminated candidiasis

We analyzed the 90-day mortality of 61 patients with CDC (Table 3). A total of 12 patients (19.7%) died within 90 days. A univariate analysis using the Kaplan–Meier method revealed that both the type of hematological disease (*P* < 0.001) and initial antifungal agents (*P* < 0.001), and timing of allogeneic transplantation (*P* < 0.001) influenced 90-day survival. Age, gender, underlying diabetes, hepatitis B, neutropenia, and thrombocytopenia were not associated with 90-day survival. A multivariate analysis with a Cox hazard regression revealed that shock was the only independent prognostic factor of 90-day survival in patients with CDC (*P* = 0.016, odds ratio < 0.001, 95% CI < 0.001–0.184).

**Table 3:** Kaplan–Meier analysis of prognostic factors of 90-day survival in 61 patients with chronic disseminated candidiasis

	Total (*n* = 61)	Alive (*n* = 49)	Death (*n* = 12)	Univariate p	Multivariate p
Age				0.539	
Age > =65 years	9	8	1		
Age < 65 years	52	41	11		
Gender				0.427	
Men	32	27	5		
Women	29	22	7		
Hematological subtype				< 0.001	0.225
AML	38	34	4		
ALL	13	8	5		
Lymphoma	7	5	2		
CML/MPN	1	1	0		
CLL	0	0	0		
Myeloma	1	1	0		
MDS/SAA	1	0	1		
Preceding allogeneic transplantation				< 0.001	0.128
Yes	5	1	4		
No	56	48	8		
Neutropenia<=500 / mm^3^				0.650	
Yes	19	16	3		
No	42	33	9		
Thrombocytopenia				0.905	
Platelet > = 20000 / mm^3^	52	42	10		
Platelet < 20000 / mm^3^	9	7	2		
Proven or Probable CDC				0.492	
Yes	16	12	4		
No	45	37	8		
Initial anti-fungal agent				< 0.001	0.624
Amphotericin B	9	7	2		
Fluconazole	35	29	6		
Itraconazole	1	0	1		
Voriconazole	6	6	0		
Posaconazole	2	1	1		
Caspofungin	1	0	1		
Micafungin	3	3	0		
Anidulafungin	2	1	1		
none	2	2	0		
Hepatitis B carrier				0.186	
Yes	7	7	0		
No	54	42	12		
Diabetes mellitus				0.854	
Yes	9	7	2		
No	52	42	10		
Shock				< 0.001	0.016
Yes	8	0	8		
No	53	49	4		

*Abbreviations*: *AML* acute myeloid leukemia, *ALL* acute lymphoblastic leukemia, *CML* chronic myeloid leukemia, *MPN* myeloid proliferative neoplasm, *CLL* chronic lymphoid leukemia, *MDS* myelodysplastic syndrome, *SAA* severe aplastic anemia, *CDC* chronic disseminated candidiasis

## Discussion

In this study, 51 (6.3%) of 808 patients with acute leukemia were diagnosed with CDC. This finding is consistent with that of our previous study, which revealed that 37 of 500 (7.4%) patients with acute leukemia developed CDC. Despite recent advances in the use of pre-emptive or prophylactic antifungals, the incidence of CDC has not decreased compared with earlier studies (incidence: 2.0–7.4%) [5, 6, 11–15]. The incidence of CDC in patients with hematological malignancies varied depending on the subtype; it was higher among patients with ALL and AML but was lower among those with CML/MPN, lymphoma, MDS/SAA, or multiple myeloma, and no CDC was recorded in patients with CLL. Most importantly, CDC was not limited to patients with acute leukemia. Other clinical characteristics such as younger age, and allogeneic transplantation were associated with a higher risk of developing hepatosplenic microabscesses.

The resolution of abscesses in imaging studies is crucial for optimizing patient care. IDSA guidelines state that therapy should continue until the lesions are resolved on repeat imaging, which is usually after several months. The premature discontinuation of antifungal therapy can lead to relapses [16]. However, studies on abscess resolution in serial imaging studies are limited. Berlow et al. reported that 2 patients showed abscess resolution in CT imaging studies after 5 and 9 months [20]. Kauffman et al. reported abscess resolution in imaging studies from 4 weeks to approximately 4–5 months, and 1 patient showed a persistent abscess for 13 months [21]. Görg et al. reported that of 6 patients with CDC, 5 had resolved lesions at 2–6 months, and 1 had a persistent lesion for more than 36 months, as demonstrated by ultrasound images [22]. In this study, 12 of 40 patients (30.0%) showed abscess resolution in imaging studies at 3 months, and 19 of 35 patients (54.3%) showed abscess resolution at 6 months. Five of 61 patients (8.2%) had persistent residual abscesses for more than 1 year. Half of patients had lesions after more than 6 months. Therefore, we suggest that imaging should be performed for most patients with CDC at 3–6 monthly intervals with at least six months of follow-up to guide therapy duration, with prolonged follow-up duration (beyond one year for certain patients with residual lesions at 6 months).

No consensus has been reached on the required duration of antifungal treatment. Parenteral antifungal agents are usually necessary at the time of diagnosis and are substituted with oral agents thereafter (fluconazole, itraconazole, voriconazole, and posaconazole). In this study, the median duration of antifungal treatment was 96 days (range: 7–796 days). As the antifungal agent administered was heterogeneous and the study retrospective in nature, the comparative therapeutic effect was difficult to determine. However, half of the abscesses on imaging studies resolved at 6 months. A treatment course of 3–6 months is warranted if lesions persist in serial imaging studies. When the factors associated with the resolution of CDC were analyzed, only recovery of neutropenia was correlated with the rapid resolution of CDC in imaging studies. Nineteen patients had neutropenia at the time of CDC diagnosis; patients whose neutropenia subsequently recovered had significantly faster imaging documented resolution than patients whose neutrophil counts did not recover (*p* < 0.001). This result implicates recovery of neutropenia is associated with rapid resolution of the microabscesses. By contrast, the hepatosplenic or renal abscesses of patients without neutropenia at the time of CDC diagnosis recovered less rapidly since the underlying immune defects predisposing to invasive fungal infections may be less easily reversed than by recovery of absolute neutrophil counts. Shoham et al. proposed that the propensity for the persistence of the fungus in infected tissues may be a consequence of cell-mediated immune dysregulation with the suppression of Th1 and the overexpression of Th2 responses [23]. The mechanisms underlying the clearance of CDC requires further investigation.

Diagnostic accuracy is a major problem in CDC. CDC without documented candidemia often remains unrecognized [24]. The mannan antigen and anti-mannan antibody have been studied as biomarkers for diagnosing CDC in patients with ALL [25]. Evidence-based reviews of currently available nonculture-based diagnostic markers of invasive candidiasis in patients with hemato-oncological malignancies are scant, and studies on the use of mannan antigen and anti-mannan antibody tests are limited to reports by the European Conference on Infections in Leukemia (ECIL) [26]. For mannan antigen and anti-mannan antibody tests, the optimal cutoffs for positivity, as well as the number of samples required for confirmation of positivity, requires validation [27]. Although the EORTC reached a consensus on the diagnosis of CDC [9, 10], the low rate of proven cases by tissue pathology or culture, low blood culture yield rates, prohibitive radiation effects of CT, severely impacts diagnostic accuracy. A recent study showed that small splenic abscesses on fluorodeoxyglucose positron emission tomography (FDG-PET) images could be missed on corresponding CT images [27]. Whether FDG-PET improves diagnostic accuracy over CT and MR imaging also requires further clarification.

This study has several limitations that are also present in clinical practice. This was a retrospective study that analyzed cases of CDC between 2008 and 2013, and the initial antifungal agents administered were heterogeneous. In this study, fluconazole was the was most commonly used first-line antifungal (57.3%), followed by amphotericin B (14.8%). The multivariate analysis revealed no significant outcome differences between the first-line antifungal agents. In this study, antifungal treatment was administered according to local guidelines in Taiwan [18, 19]. Several international guidelines have been developed for invasive candidiasis and antifungal prophylaxis and treatment in patients with hematological malignancies [16, 28, 29]. Accordingly, the Taiwan National Health Insurance provided reimbursement since July 2015 for posaconazole used in the context of antifungal prophylaxis among patients with AML in Taiwan. The incidence of CDC after this secular change is an ongoing study.

The univariate analysis revealed that hematological subtypes, preceding allogeneic transplantation, and administration of initial antifungal agents were poor prognostic factors of CDC. The multivariate analysis indicated that shock was an independent prognostic factor of CDC. In total, 22 patients underwent allogeneic transplantation after the infection was controlled. CDC was not an absolute contraindication to stem cell transplantation [14, 30] since confirmation of invasive candidiasis represents a major diagnostic and therapeutic challenge for physicians [31]. Early intervention strategies based on clinical prediction rules and nonculture-based diagnostic assays may improve the outcomes of these healthcare-associated complications.

## Conclusion

The incidence of chronic disseminated (hepatosplenic/renal) candidiasis did not decrease between 2008 and 2013. Patients with acute leukemia have a higher but not exclusive risk of CDC than those with other hematological malignancies. Repeated imaging studies at six months revealed that the hepatosplenic microabscesses had resolved in only half of the patients and it was necessary to continue therapy beyond six months for the remaining half. According to our findings, CDC requires prolonged treatment and follow-up. Among all criteria, shock is the only independent prognostic factor of 90-day survival in patients with CDC.

## Data Availability

The datasets generated and analyzed during the current study are not publicly available without application to the Research Ethics Committee of National Taiwan University Hospital.

## Abbreviations

ALL: Acute lymphoblastic leukemia
AML: Acute myeloid leukemia
CDC: Chronic disseminated candidiasis
CLL: Chronic lymphocytic leukemia
CML: Chronic myeloid leukemia
CT: Computed tomography
ECIL: European Conference on Infections in Leukemia
EORTC: European Organization for Research and Treatment of Cancer
FDG-PET: Fluorodeoxyglucose positron emission tomography
IDSA: Infectious Diseases Society of America
MDS: Myelodysplastic syndrome
MM: Multiple myeloma
MPN: Myeloproliferative neoplasm, SAA: severe aplastic anemia
